# A Phylogenetic View on the Role of Glycerol for Growth Enhancement and Reuterin Formation in *Limosilactobacillus reuteri*

**DOI:** 10.3389/fmicb.2020.601422

**Published:** 2020-12-21

**Authors:** Zhihong Zhang, Kaiming Wang, Jee-Hwan Oh, Shenwei Zhang, Jan-Peter van Pijkeren, Christopher C. Cheng, Dayong Ren, Hua Wei, Michael G. Gänzle, Jens Walter

**Affiliations:** ^1^State Key Laboratory of Food Science and Technology, Nanchang University, Nanchang, China; ^2^Agricultural, Food and Nutritional Science, University of Alberta, Edmonton, AB, Canada; ^3^Department of Physiology, CEGIIR, University of Alberta, Edmonton, AB, Canada; ^4^Department of Food Science, University of Wisconsin-Madison, Madison, WI, United States; ^5^College of Food Science and Engineering, Jilin Agricultural University, Changchun, China; ^6^Department of Biological Sciences, University of Alberta, Edmonton, AB, Canada; ^7^APC Microbiome Ireland, School of Microbiology, University College Cork, Cork, Ireland; ^8^Department of Medicine, University College Cork, Cork, Ireland

**Keywords:** *Limosilactobacillus reuteri*, reuterin, glycerol, carbohydrate utilization, growth rate, reuterin resistance

## Abstract

Lineages within the species *Limosilactobacillus reuteri* have specialized to various hosts and their genomes reflect these adaptations. The *pdu-cbi-cob-hem* gene cluster is conserved in most human and poultry isolates but is infrequent in rodent and porcine isolates. This gene cluster confers the transformation of glycerol into 3-hydroxy-propionaldehyde (reuterin), which can either be secreted and function as precursor of the antimicrobial compound acrolein or serve as an electron acceptor that enhances the organisms’ growth rate. However, it remains unclear which of these two functions is more relevant for *L. reuteri* evolution and ecology. Here we characterized the effect of glycerol on growth rate and reuterin formation in *L. reuteri* strains across different phylogenetic lineages during growth on ecologically relevant carbohydrates. We further evaluated the innate reuterin resistance among these strains to infer a possible role of reuterin in the evolution of strains. Results revealed that the poultry/human lineage VI strain, *L. reuteri* DSM 17938 shows more growth enhancement through glycerol and greater capacity for reuterin production on glucose and maltose as compared to human lineage II strains. Interestingly, reuterin production in lineage II strains was significantly elevated on raffinose and lactose, reaching levels similar to DSM 17938. On all carbohydrates tested, reuterin production occurred during the exponential growth phase and became undetectable during the stationary growth phase. The amount of reuterin produced was sufficient to inhibit *E. coli*, suggesting that it could be ecologically relevant, but the resistance towards reuterin among *L. reuteri* strains was highly variable and, for the most part, unrelated to the strain’s capacity for reuterin production. Overall, the findings suggest differences in the substrate-specific regulation of the *pdu* cluster in *L. reuteri* lineages that might be reflective of their ecological niches, e.g., chicken foregut versus human infant and adult large intestine. Such information can inform future studies on the ecology of *L. reuteri* and guide the development of synbiotic applications to improve the therapeutic use of this species.

## Introduction

*Limosilactobacillus reuteri* (formerly *Lactobacillus reuteri*, Zheng et al., 2020) inhabits the gut of vertebrate animals and has diversified into host specific phylogenetic lineages whose gene content reflects adaptation to their particular hosts (Walter et al., 2011; Duar et al., 2017). One of the gene clusters associated with *L. reuteri* differentiation into divergent phylogenetic lineages is the *pdu-cbi-cob-hem* cluster (*pdu* cluster). This cluster is conserved within the lineage II (human/herbivore), and lineage VI (poultry/human) strains but infrequently occurs in rodent isolates (lineages I and III) (Frese et al., 2011; Walter et al., 2011). The *pdu* cluster was likely acquired by horizontal gene transfer early in the evolution of *L. reuteri* (Morita et al., 2008) but appears to have been deleted in most lineages in response to the host environment while being retained in the human and poultry lineages (Frese et al., 2011). This distribution suggests a possible role of the cluster in the host adaptation process of *L. reuteri*.

The *pdu* cluster serves at least two functions. First, it allows the organism to utilize 1,2-propanediol and glycerol as electron acceptors, facilitating the recovery of an extra ATP and enhancing growth (Luthi-Peng et al., 2002; Dishisha, 2014; Gänzle, 2015; Cheng et al., 2020); Additionally, it converts glycerol to metabolic intermediate β-hydroxy-propionaldehyde, which is reuterin in solution and forms the cytotoxic and antimicrobial compound acrolein (Talarico et al., 1988; Engels et al., 2016b; Fernandez-Cruz et al., 2016). The functional role of reuterin in gut ecosystems remains poorly defined. Reuterin produced by *L. reuteri* inhibits other bacteria, and *L. reuteri* is more resistant towards reuterin than most other intestinal bacteria, which suggests that the antibacterial activity of reuterin may confer ecological and evolutionary significance (Cleusix et al., 2007; De Weirdt et al., 2012; Spinler et al., 2017). However, the production of reuterin requires high concentrations of glycerol, and in the presence of glucose, reuterin is rapidly reduced to propanediol (Luthi-Peng et al., 2002). Intestinal glycerol metabolism generates acrolein, but given its reactivity, it is unclear if the compound exerts antimicrobial activity *in vivo* (Zhang J. et al., 2018). Although gene contents and organization within the *pdu* cluster are conserved among strains, lineage-specific sequence differences lead to a distinct gene expression profile and reuterin production among human/herbivore lineage II and poultry/human lineage VI strains, suggesting divergent roles of the cluster in different host-adapted lineages (Spinler et al., 2014). However, the evolutionary and ecological role of the *pdu* cluster has not been elucidated, and it remains unclear which of the two functions (growth enhancement and reuterin formation) determine the relevance of the cluster in this context. This question is especially relevant as the *pdu* cluster constitutes a fitness burden on *L. reuteri* in the mouse gut in absence of relevant substrates (Cheng et al., 2020).

*Limosilactobacillus reuteri* lineages occupy different intestinal niches specific to their host species, which is characterized by biofilm formation in the proximal gastrointestinal tract in animals such as chicken (crop), pigs (pars esophagus), and rodents (forestomach). Such epithelial associations have not been described for human strains, which lack most of the adhesins and biofilm-related genes identified in rodent strains (Frese et al., 2013; Krumbeck et al., 2016). The carbohydrates available for growth also differ in their respective niches occupied by *L. reuteri* (Talarico et al., 1990; Zhao and Gänzle, 2018). Mono- and disaccharides, including maltose, sucrose, and fructose, are readily available in the proximal gut of chicken and rodents but are scarce in the human colon owing to the prior absorption in the small intestine. In comparison, indigestible dietary carbohydrates, such as raffinose, may serve as growth substrates for gut microbes in the large intestine (Zartl et al., 2018). Lactose is also available as a carbon source in the intestine of suckling mammals, and lactobacilli are among the main lactose metabolizing organisms in the swine hindgut (Wang et al., 2019). To our knowledge, glucose is currently the sole sugar that has been studied regarding the functionality of the *pdu* cluster, demonstrating that reuterin formation is favored under low glucose concentrations (El-Ziney et al., 1998; Luthi-Peng et al., 2002). However, maltose, sucrose, and raffinose rather than glucose are more likely to be available in gut ecosystems and are the preferred substrates for *L. reuteri* (Zhao and Gänzle, 2018). Thus, the functions encoded by the *pdu* cluster have not been studied when *L. reuteri* strains have been grown with substrates that are ecologically relevant.

The goal of this study was to examine glycerol-related functions of the *pdu* cluster in representative *L. reuteri* strains originating from different phylogenetic lineages using relevant growth substrates. We evaluated both growth rate enhancement and reuterin formation in the presence of glycerol and relevant concentrations of either glucose, maltose, lactose, or raffinose as the carbohydrate source. We further integrated this data with information on reuterin resistance among these strains.

## Materials and Methods

### Strains, Media and Culture Condition

*Limosilactobacillus reuteri* strains used in this study are listed in Table 1, along with their respective phylogenetic lineages (Oh et al., 2010). Eight strains contain the *pdu* cluster (Morita et al., 2008; Frese et al., 2011; Walter et al., 2011), five strains without the *pdu* cluster, and two isogenic mutants of *L. reuteri* ATCC 6475 and *L. reuteri* DSM 17938 with an inactivated *pdu* cluster (ATCC 6475Δ*pduCDE* and DSM 17938Δ*pduC*, respectively). *L. reuteri* strains were grown in Difco^TM^ Lactobacilli MRS broth or on agar plates containing 1.5% agar at 37°C in an anaerobic chamber (GENEQ inc., BACTRON^TM^, United States) supplied a gas mix of 5% CO_2_, 5% H_2_ and 90% N_2_. *Escherichia coli* cultured in Luria-Bertani (LB) medium for aerobic growth at 37°C.

**TABLE 1:** *L. reuteri* strains used in this study.

Strain	Origin	Lineage	*pdu* cluster	Source^a^
Lpuph-1	Rodent	I	No	(Oh et al., 2010)
DSM 20056	Rodent	I	No	(Oh et al., 2010)
mlc3	Rodent	III	Yes	(Oh et al., 2010)
N2D	Rodent	III	No	(Oh et al., 2010)
DSM 20016^T^	Human	II	Yes	(Oh et al., 2010)
ATCC 6475^b^	Human	II	Yes	(Oh et al., 2010)
ATCC 6475Δ*pduCDE*	ATCC 6475	II	Inactivated	(Zhang S. et al., 2018)
PB-W1	Human	na^c^	No	This study
LPA1	Pig	IV	Yes	(Oh et al., 2010)
ATCC 53608	Pig	IV	Yes	(Oh et al., 2010)
jw2015	Pig	IV	Yes	(Oh et al., 2010)
20-2	Pig	V	No	(Oh et al., 2010)
CF48-3A1	Human	VI	Yes	(Oh et al., 2010)
DSM 17938	Human	VI	Yes	(Oh et al., 2010)
DSM 17938Δ*pduC*	DSM 17938	VI	Inactivated	This study

*^*a*^Further information on the listed source sequences can be found at the Joint Genome Institute (JGI) genome portal (http://genome.jgi.doe.gov); ^*b*^The name of ATCC 6475 is mm4-1a in the report (Oh et al., 2010); ^*c*^not assigned.*

### Construction of Mutant *L. reuteri* Strains

*Limosilactobacillus reuteri* ATCC 6475Δ*pduCDE* was constructed previously (Zhang S. et al., 2018). To generate a *pdu* cluster inactivated *L. reuteri* DSM 17938, we deleted the gene coding for the large subunit of the glycerol dehydratase (*pduC*) using a recently developed counter selection system (Zhang S. et al., 2018). First, we amplified the upstream and downstream regions by PCR, 750 bp each, flanking the *pduC* gene using oligonucleotide pairs oVPL2056-oVPL2057 and oVPL2058-oVPL2059 (Table 2), respectively. The backbone of the integration vector (pVPL3002) was amplified with oligonucleotide pair oVPL187-oVPL188. The three amplicons were fused by ligation cycle reaction (LCR) with three bridging oligonucleotides (oVPL2060, 2061, and 2062) (De Kok et al., 2014), and transformed in electro-competent *E*. *coli* EC1000 (Green and Sambrook, 2012). By colony PCR (oVPL49-oVPL97), insertion of the upstream and downstream flanking regions was confirmed. The integrity of the cloned sequence was confirmed by Sanger sequencing and the resultant construct was named pVPL3771. Next, *L. reuteri* DSM 17938 harboring the helper-plasmid pVE6007 (Law et al., 1995) was transformed with pVPL3771, and cells were recovered for 48 h in MRS harboring 5 μg/mL chloramphenicol and erythromycin. Cells were washed and subcultured (0.1% v/v) in MRS containing erythromycin (5 μg/mL). Cells were incubated for 20 generations to facilitate the loss of pVE6007 and integration of pVPL3771, followed by 10 generations growth in absence of antibiotics to allow a second homologous recombination event to occur. Bacteria were plated on MRS plates containing vancomycin (500 μg/mL). Only bacteria which have lost pVPL3771 will be recovered, *i.e.*, have undergone a second homologous recombination event. By colony PCR with primers oVPL2063-oVPL2064 we identified cells with the mutant genotype and confirmed deletion of *pduC*. The resultant strain was named *L. reuteri* DSM 17938Δ*pduC*.

**TABLE 2:** The oligonucleotides used for *pduC* deletion of DSM 17938.

Characterization	Oligonucleotide	Sequence (5′→ 3′)
u/s flanking Fwd^a^	oVPL2056^c^	ctccttaatcgaacgtaactttg
u/s flanking Rev	oVPL2057	gaacacttggaactatagagagg
d/s flanking Fwd^b^	oVPL2058	acgcttcataataaagtagtcctc
d/s flanking Rev	oVPL2059	atgaatgactttctaaattctactagc
pVPL3002 Backbone Fwd^c^	oVPL187	taccgagctcgaattcactgg
pVPL3002 Backbone Rev	oVPL188	atcctctagagtcgacctgc
Bridging oligo1	oVPL2060	aaacgacggccagtgaattcgag ctcggtactccttaatcgaacgtaac tttgatttctt
Bridging oligo2	oVPL2061	attacctcctctctatagttccaagtgttc acgcttcataata aagtagtcctccttcca
Bridging oligo3	oVPL2062	tgtgctagtagaatttagaaagtcattc atatcctctagagtcgacctg caggcatgcaa
DCO screen Fwd^*d*^	oVPL2063	ttgctactaaatcatcaacttcactc
DCO screen Rev	oVPL2064	tattaggagcttttataagcaggag
flanking cloning site pVPL3002	oVPL49	acaatttcacacaggaaacagc
flanking cloning site pVPL3002	oVPL97	cccccattaagtgccgagtgc

*^*a*^u/s, up-stream; ^*b*^d/s, down-stream; ^*c*^pVPLxxxx (Van Pijkeren Lab oligonucleotide collection identification number); ^*d*^DCO, double crossover.*

### Impact of Glycerol on Growth Rate in the Presence of Different Carbohydrate Substrates

Growth rates of strains were measured with or without the presence of glycerol in the media. Single colonies of *L. reuteri* strains were inoculated in MRS at 37°C for 24 h under anaerobic condition. Strains were subsequently sub-cultured twice and cultures were used to inoculate (1.0% inoculums, v/v) fresh basal MRS medium (10 g proteose peptone, 5 g beef extract, 5 g yeast extract, 3 g NH_4_Cl, 4 g K_2_HPO_4_, 2.6 g KH_2_PO_4_, 0.05 g MgSO_4_, 0.05 g MnSO_4_⋅4H_2_O, 0.5 g L-cysteine hydrochloride, and 1.0 g Tween 80 per liter) supplemented with 0.5% (wt/vol) of either glucose, maltose, lactose or raffinose in the presence or absence of 50 mM glycerol. Growth was determined in volumes of 200 μL in 96-well microtiter plates (Nunclon Delta Surface, Thermo Scientific), with each well covered with 45 μL mineral oil to maintain anaerobic condition. Plates were incubated at 37°C and OD_600_ values relative to uninoculated controls were monitored using a spectrophotometer (SpectraMax M3, CA, United States) hourly for 16 h. Experiments were performed in triplicate, and the growth rates were estimated using the following formula: μ = (lnOD_2_-lnOD_1_)/(t_2_-t_1_), where OD_1_ = optical density at onset of the exponential phase of growth (t_1_) and OD_2_ = optical density at the end of the exponential phase of growth (t_2_) (Witkowska et al., 2017).

### Determination of Reuterin Formation

Reuterin formation by *L. reuteri* strains was assessed as described by Spinler et al. (2014). In brief, cell pellets from overnight cultures were washed with 0.01 M phosphate buffered saline (pH 7.4) and resuspended to ∼1.5 × 10^9^ cfu/mL in 250 mM glycerol solution supplemented with 0.5% (wt/vol) of either glucose, maltose, lactose or raffinose. Solutions were incubated anaerobically for 2 h at 37^o^C, and supernatants were obtained using centrifugation at 12, 000 × *g* for 5 min and filter-sterilized with a 0.22 μm polyvinylidene fluoride membrane filter (Millipore, United States) and stored at 4°C. Reuterin was quantified in 10-fold diluted supernatants using a colorimetric method as described (Spinler et al., 2008). Briefly, supernatants (50 μL) were mixed with 37.5 μL tryptophan (0.01 M solution in 0.05 M HCl) followed by addition of 150 μL concentrated HCl immediately, and 200 μL was added to 96 well microtiter plates and incubated at 37°C for 30 min. The negative control was performed with supernatants being replaced by double distilled H_2_O. The absorbance of each well was subsequently measured under 560 nm using a spectrophotometer (SpectraMax M3, CA, United States). Reuterin was quantified using a standard curve generated from a serial 2-fold diluted acrolein (Doleyres et al., 2005).

### Determination of Reuterin Formation Using an Inhibition Plate Assay

*Limosilactobacillus reuteri* strains ATCC 6475 and DSM 17938 as well as their cognate *pdu* negative mutants were used to determine reuterin production on an inhibition spot plate assay with *E. coli* MG1655 as the indicator organism. Assays were performed using an agar spot method as described by Spinler et al. (2017) with modifications. In brief, overnight cultures of *L. reuteri* (2 μL) were spotted on basal MRS agar supplemented with 1.5% of different carbohydrates (glucose, maltose, lactose, or raffinose) and grown in anaerobic chamber (GENEQ inc, BACTRON^TM^, United States) at 37°C for 12 h. Agar plates were then overlaid with soft agar (LB medium, 250 mM glycerol, 0.7% agar) containing 1.0% carbohydrate (glucose, maltose, lactose or raffinose) and 10^5^ ∼ 10^6^ cfu/mL overnight culture of *E. coli* MG1655 and incubated aerobically at 37°C for 16 h. Another condition with 1.0% carbohydrate in basal MRS agar and 1.0% carbohydrate plus 300 mM glycerol in soft agar was also performed. Negative controls were processed using the same method, but without addition of glycerol. Inhibition was quantified by halo size.

### Reuterin Formation During Different Growth-Phases

To characterize reuterin production during growth with different carbohydrate substrates, strains DSM 17938 and ATCC 6475 were sub-cultured twice in broth, and overnight cell cultures (1.0%) were inoculated to basal MRS supplemented with 0.5% of either glucose, maltose, lactose or raffinose and 50 mM glycerol, and incubated anaerobically at 37°C. Samples (1 mL) were collected at 4, 6, 8, 10, and 12 h, and OD_600_ values and reuterin concentrations were determined through colorimetric assays as described above. Reuterin production from mutant strains DSM 17938Δ*pduC* and ATCC 6475Δ*pduCDE* were also tested as negative controls.

### Determination of Resistance of *L. reuteri* Strains to Reuterin

Resistance of *L. reuteri* strains to reuterin was tested by growth inhibition assays conducted in 96-well microtiter plates. Aliquots of 100 μL basal MRS containing 0.5% (m/v) of either glucose, maltose, lactose or raffinose were added to each well of the plates, and 200 μL supernatant from DSM 17938 fermentation with 250 mM glycerol and 0.5% glucose as described above were added in first well, then a 1:3 serial dilution was performed using all wells of the plate, while the supernatant from DSM 17938Δ*pduC* fermentation served as a negative control. Overnight cell cultures (1.0%) were inoculated to each well, and each well was covered with 45 μL mineral oil. The plates were incubated at 37°C and OD_600_ values were measured using a spectrophotometer at 0 h and 16 h. Inhibition was expressed as MIC_50_ which was read as the lowest concentration that reduced 50% of growth compared to positive control.

### Statistical Analyses

Data was analyzed with GraphPad Prism 5.0 (GraphPad Software, Inc., CA, United States). Differences were determined with two-way analysis of variance (ANOVA) test and *p* < 0.05 indicated significant differences. All experiments were performed as triplicate biological replicates.

## Results

### The *pdu* Cluster Allows for Glycerol Dependent Growth Enhancement in *L. reuteri*

**FIGURE 1:**
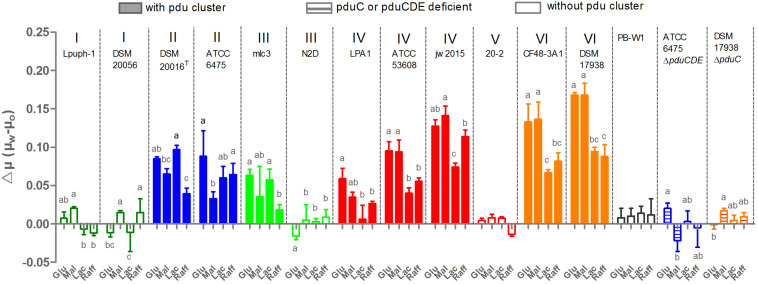
Growth rates enhancement of *L. reuteri* strains through the presence of glycerol in the media. μ_w_ and μ_o_ represent growth rates with and without the presence of glycerol, respectively; Glu, Mal, Lac, Raff indicate glucose, maltose, lactose and raffinose, respectively; Data represent means ± standard deviations. Data for the same strain grown on different sugars differ significantly (*p* < 0.05) if bars don’t share a common superscript.

**FIGURE 2:**
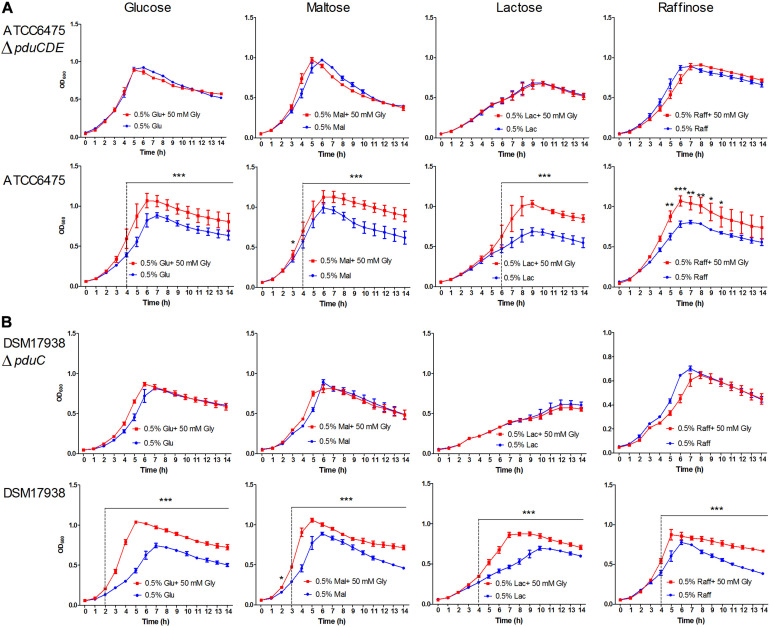
Growth curves of representative strains with and without glycerol addition in the media. **(A)** ATCC 6475Δ*pduCDE* vs ATCC 6475; **(B)** DSM 17938Δ*pduC* vs DSM 17938. Data represent means ± standard deviations with and without glycerol, significant differences are indicated: *p < 0.05; **p < 0.01; ***p < 0.001.

The capacity for glycerol to enhance growth in *pdu* cluster containing *L. reuteri* species has been shown previously using glucose as the substrate, with the *pdu* cluster facilitating glycerol dehydration to reuterin (β-hydroxy-propionaldehyde) and its subsequent reduction to 1,3-propanediol (El-Ziney et al., 1998; Engels et al., 2016b). This reduction is accompanied by NADH reoxidation and stimulates growth by yielding additional ATP production from the phosphoketolase pathway (Talarico et al., 1990; Chen et al., 2016). To characterize the functional significance of the *pdu* cluster in a phylogenetic context, we assessed the acceleration of growth by glycerol in 15 *L. reuteri* strains across six lineages using the carbohydrate sources glucose, maltose, lactose, and raffinose. The results revealed that the ability to utilize glycerol for enhancing growth rate was strictly dependent on the *pdu* cluster. Glycerol addition did not enhance growth of wild-type strains without the *pdu* cluster, or of mutants with deletions in the *pdu* cluster (Figure 1). Moreover, the growth rate enhancement further differed amongst strains across different phylogenetic lineages. Lineage IV (porcine) and lineage VI (poultry/human) strains benefitted from glycerol to a larger extent when grown with glucose and maltose as compared to lactose and raffinose (*p* < 0.05). Such substrate-specific differences were not observed in lineage II (human) and lineage III (rodent) strains, which also showed an overall lower degree of growth rate enhancement relative to lineage VI strains on glucose and maltose (*p* < 0.01). Based on the detailed growth curves, wild-type ATCC 6475 and DSM 17938 clearly showed a significant growth advantage with glycerol as compared to without glycerol as early as 2 h (*p* < 0.05). Growth enhancement was completely absent in strains that do not possess the *pdu* as well as strains in which the cluster was inactivated (ATCC 6475Δ*pduCDE* and DSM 17938Δ*pduC*) (Figures 1, 2). Thus, our growth analysis supports the *pdu* cluster’s role in *L. reuteri* utilization of glycerol to enhance growth, while showing lineage-specific differences based on carbohydrate sources. Lineage VI (CF48-3A1 and DSM 17938) strains benefited from a significantly higher growth enhancement with glucose and maltose compared to lactose or raffinose (*p* < 0.01; *p* < 0.01, respectively), whereas the growth enhancement in lineage II strains (DSM20016^T^ and ATCC 6475) was generally lower and much less dependent on carbohydrate source.

### Differential Substrate-Specific Regulation of Reuterin Biosynthesis Among Lineages

**FIGURE 3:**
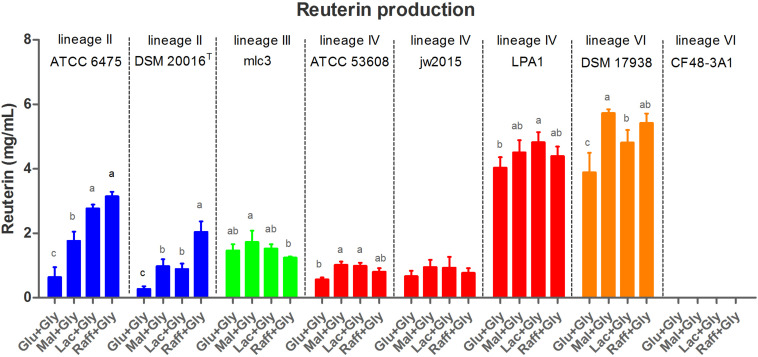
Reuterin concentrations in supernatant of *L. reuteri* fermentations with 250 mM glycerol buffer and 0.5% (wt/vol) carbohydrate. Cell numbers were standardized to ∼1.5 × 10^9^ cfu/mL. Glu, glucose; Mal, maltose; Lac, lactose; Raff, raffinose; and Gly, glycerol. Data for the same strain grown on different sugars differ significantly (*p* < 0.05) if bars don’t share a common superscript.

To explore the role of the *pdu* cluster in reuterin production, the same set of *L. reuteri* strains was used to characterize reuterin formation during glycerol fermentation. Reuterin production in the supernatant was quantified using the standard curve of acrolein (Supplementary Figure 1). As expected, wild-type strains without the *pdu* cluster and the two mutant strains ATCC 6475Δ*pduCDE* and DSM 17938Δ*pduC* failed to produce reuterin (Figure 3). All wild-type strains with the *pdu* cluster (except CF48-3A1) produced reuterin but showed differences based on phylogenetic lineages and the carbohydrate sources (Figure 3). Lineage VI human-derived DSM 17938 and lineage IV porcine-derived LPA1 demonstrated the highest reuterin production levels, with comparable amounts produced across all four carbohydrates (although production was significantly lower with glucose in DSM 17938, *p* < 0.05). In comparison, lineage III mlc3 and lineage IV ATCC 53608 and jw2015 produced relatively lower quantities of reuterin regardless of the carbohydrate source (*p* < 0.001). Notably, in lineage II ATCC 6475 and DSM 20016^T^, reuterin production was almost completely repressed with glucose, while production with raffinose was significantly enhanced (*p* < 0.001), with maltose and lactose yielding intermediate concentrations of reuterin. Although glucose did also appear to repress reuterin formation in DSM 17938, the effect was not as pronounced as in lineage II strains.

### Substrate Specific Regulation in Lineage II ATCC 6475 Is Also Detectable in Inhibition Assays

**FIGURE 4:**
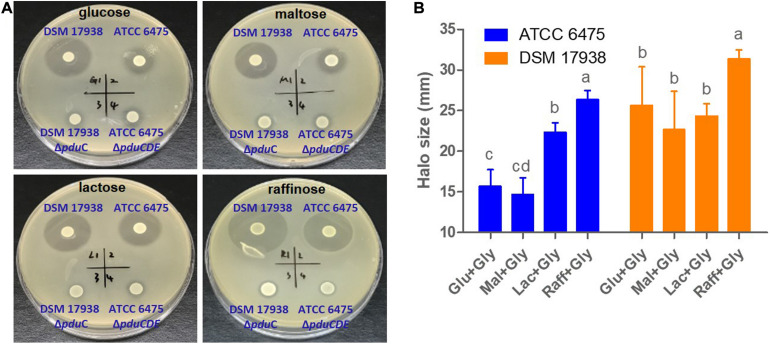
Carbohydrate dependent reuterin production. Spot plate assays of lineage II human strain ATCC 6475, lineage VI strain DSM 17938, and their respective *pdu* mutants were spotted on basal MRS with 1.5% carbohydrate before being overlaid with soft agar containing *E. coli* MG1566 as well as 250 mM glycerol with 1.0% carbohydrate. **(A)** Representative images showing halos of inhibition for the wild-type strains. **(B)** Halo sizes in relation to carbohydrate source. Data represent means ± standard deviations. Data for the same strain grown on different sugars differ significantly (*p* < 0.05) if bars don’t share a common superscript.

To confirm that the substance detected using the colorimetric assay was indeed reuterin, and to validate the substrate-specific differences in reuterin production between lineages II and VI strains, we compared the antimicrobial activity exerted by *L. reuteri* strains ATCC 6475, DSM 17938, and their respective mutants on *E. coli*. As shown in Figure 4 and Supplementary Figure 3, on either glucose or maltose, DSM 17938 resulted in much higher inhibition than ATCC 6475 (*p* < 0.001; *p* < 0.001), which is reflected through the larger sized halos on soft agar. Although raffinose led to a slightly higher production of reuterin in DSM17938 (Figure 4), there were no major differences among sugars. In contrast, inhibition of ATCC 6475 was substantially higher when grown on lactose and raffinose as compared to glucose or maltose (Figure 4 and Supplementary Figure 3; *p* < 0.05) and reached similar values as those for DSM 17938 when grown with raffinose. As expected, no inhibition was detected for mutant strains, and for both wild type and mutant strains without the addition of glycerol to the soft agar (Supplementary Figure 4), confirming that the inhibitory effect on *E*. *coli* was exerted by reuterin and not other metabolites. Overall, these inhibition experiments confirmed our findings above obtained with glycerol fermentation, suggesting that extent of reuterin formation, and its regulation in relation to carbohydrate sources, differs among strains. In ATCC 6475, but not in DSM 17938, reuterin formation is almost completely repressed with glucose and maltose and much lower as compared to DSM 17938, while with raffinose, both strains produce comparable amounts.

### Reuterin Is Produced During the Exponential Phase of Growth

**FIGURE 5:**
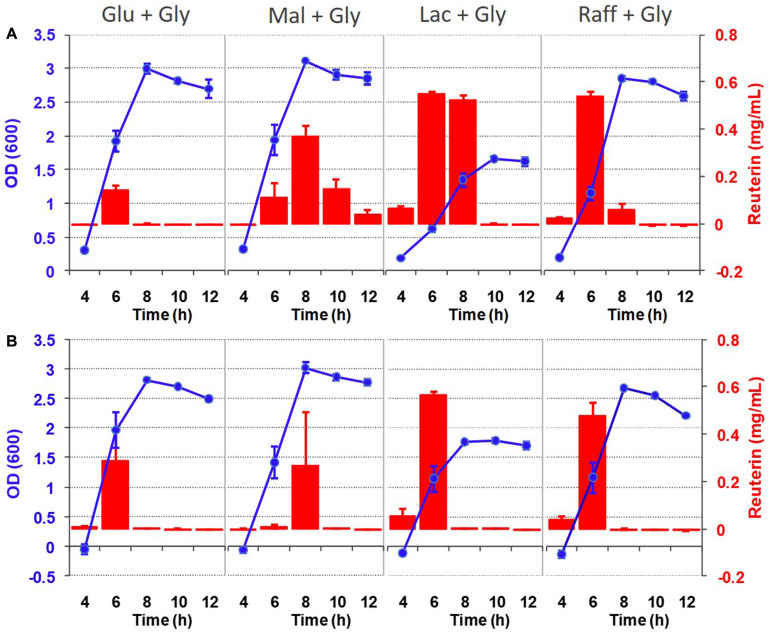
Reuterin formation during the growth of *L. reuteri* strains DSM 17938 **(A)** and ATCC 6475 **(B)**. Cells were grown in basal MRS supplemented with 0.5% carbohydrate and 50 mM glycerol at 37°C and OD_600_ and reuterin concentrations were measured at 4, 6, 8, 10, and 12 h after inoculation. Data represent means ± standard deviations of three biological replicates.

Given that the *pdu* cluster contributes to both growth and reuterin formation, we wondered how these two traits are related. Thus, we determined the temporal pattern of reuterin formation in relation to growth in basal MRS with different carbohydrates using the colorimetric assay. As shown in Figure 5, reuterin from DSM 17938 and ATCC 6475 was only detectable during the exponential growth phase. Reuterin quickly dissipated after reaching the stationary phase in both strains. As expected, no reuterin was obtained from the mutant DSM 17938Δ*pduC* and ATCC 6475Δ*pduCDE* during growth. Overall, these findings indicate that reuterin production occurred simultaneous to *L. reuteri* growth, indicating that glycerol is simultaneously used to support growth and the formation of reuterin.

### Reuterin Resistance Is Strain Dependent and Not Linked to Production

**FIGURE 6:**
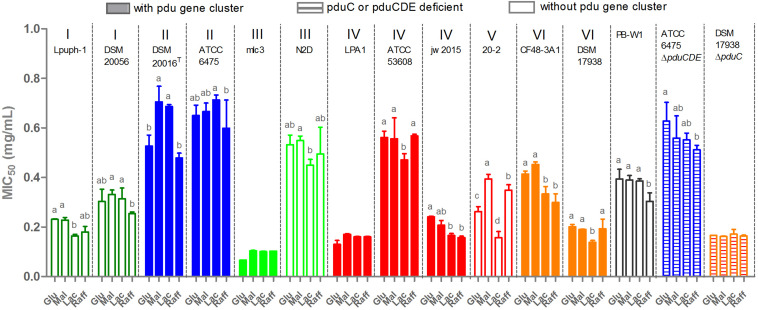
Reuterin resistance of *L. reuteri* strains from some different hosts. Glu, glucose; Mal, maltose; Lac, lactose; and Raff, raffinose. MIC_50_ was calculated by Graphpad Prism 7.0 and results present as means ± standard deviations of three biological replicates. Data for the same strain grown on different sugars differ significantly (*p* < 0.05) if bars don’t share a common superscript.

Given the previous findings that *L. reuteri* is more resistant towards reuterin than other common gut bacteria (Cleusix et al., 2007), and the substantial inter-strain variation in reuterin production, we hypothesized that *L. reuteri* strains with greater capacity for reuterin formation would demonstrate higher resistance towards reuterin. To test this hypothesis, we determined the MIC_50_ for all *L. reuteri* strains toward reuterin. Surprisingly, *L. reuteri* resistance to reuterin was strain-dependent and not linked to reuterin formation or phylogeny (Figure 6). For example, all three porcine-derived lineage IV strains, LPA1, ATCC 53608, and jw2015 are reuterin producers, yet ATCC 53608 demonstrated significantly higher reuterin resistance (*p* < 0.001), despite producing much less reuterin during glycerol fermentation than LPA1. Additionally, lineage VI DSM 17938, one of five human-derived strains, was extremely sensitive to its self-produced reuterin (MIC_50_ < 0.2 mg/mL), despite having the greatest reuterin producing capacity amongst all the strains tested. The other lineage VI strain CF48-3A1, although failing to produce reuterin, demonstrated much higher reuterin resistance than DSM 17938 (*p* < 0.01). The resistance of the mutants ATCC 6475Δ*pduCDE* and DSM 17938Δ*pduC* was indistinguishable from their respective wide type strain, showing that the deleted genes in the *pdu* cluster did not contribute towards reuterin resistance. In most cases, the carbohydrates supplied for growth had little effect on MICs. Additionally, no efficient inhibition of *L. reuteri* strains was found by the serially diluted supernatant from DSM 17938Δ*pduC* glycerol fermentation, indicating that reuterin is indeed the metabolite inhibiting growth. Overall, only human-derived ATCC 6475, DSM 20016^T^, and porcine-derived ATCC 53608 are reuterin producers with elevated reuterin resistance levels, while N2D and PB-W1 displayed relatively high levels of resistance despite lacking the *pdu* cluster themselves.

## Discussion

The *pdu* cluster in *L. reuteri* encodes genes for the utilization of glycerol and 1,2-propanediol to enhance growth. Additionally, it mediates the formation of a metabolite reuterin from glycerol fermentation (Luthi-Peng et al., 2002; Dishisha, 2014; Gänzle, 2015). Although functions of the *pdu* cluster have been established, the ecological role of glycerol metabolism by *L. reuteri* is unclear. Here we demonstrate that most strains possessing the *pdu* cluster benefit from glycerol-induced growth enhancement, while high inter-strain variability exists in both the ability to produce reuterin, and the resistance to reuterin. Moreover, the capacity for reuterin formation and the degree of resistance towards reuterin rarely correlates with each other. Despite the inter-strain variation in *pdu* encoded functions, growth phenotype and reuterin production displayed interesting phylogenetic relationships. For phylogenetic lineages IV and VI, growth stimulation by glycerol was higher with glucose and maltose than lactose and raffinose as the carbohydrate source, while glycerol supported growth to a much lesser extent in lineage II and III strains. Conversely, reuterin formation was especially pronounced with the substrates raffinose and lactose, especially for human-derived lineage II DSM 20016^T^ and ATCC 6475, which produced very low reuterin levels in presence of glucose. In comparison, human-derived lineage VI DSM 17938 demonstrated much greater reuterin formation capacity under glucose than DSM 20016^T^ or ATCC 6475, which agrees with previous observations by Spinler et al. (2014) that lineage VI strains produce more reuterin than lineage II strains. A novel and very relevant finding of our study, both for the interpretation of *L. reuteri* ecology and its biotechnological applications, is that human lineage II strains produce much higher quantities of reuterin with lactose and raffinose as the carbohydrate sources.

Reuterin was considered to be simply a metabolic intermediate of glycerol metabolism in *L. reuteri* (Talarico et al., 1990). Our data largely agrees with this because growing cells only transiently accumulate reuterin during the exponential growth phase. Interestingly, the concentration of reuterin (Figure 5) was approximately equivalent to the inhibitory concentration of the producer strains (Figure 6), suggesting that growth inhibition may limit production. In the stationary growth phase, reuterin is converted into 1,3-propanediol (Engels et al., 2016b). However, we also found a strong influence of the carbohydrate source on reuterin formation, particularly in human lineage II strains, which includes the type strain DSM 20016^T^ and commercially important probiotic strains. Using three different assays, we showed that raffinose and, to a lesser extent, lactose supplementation results in much higher levels of reuterin formation in lineage II strains when compared to glucose or maltose.

The metabolism of *L. reuteri* provides a potential explanation for our findings. Diol metabolism encoded by the *pdu* cluster has two alternative metabolic substrates, glycerol and 1,2-propanediol, with 1,3-propandiol or propanol, respectively, and 2-hydroxy propanoic acid or propanoic acid, respectively, as the alternative products (Gänzle, 2015; Engels et al., 2016a). The role of the pathway for metabolism and competitiveness depends on the availability of substrates and hexoses. In food and feed, glycerol as well as hexoses are available from triglycerides but also from esters of phenolic acids and other secondary plant metabolites (Zhao and Gänzle, 2018). In cereal substrates that match the substrate availability of the upper intestinal tract of chicken and rodents, maltose, sucrose and glucose are the main carbon sources. The availability of glycerol supports formation of 4-12 mmol/L 1,3-propanediol by *L. reuteri* (Lin and Ganzle, 2014), and 1,3-propandiol or propanol increases the energy yield of hexose metabolism in the phosphoketolase pathway by regenerating NADH (Gänzle, 2015; Engels et al., 2016a). Glycerol metabolism also accumulates active concentrations of the antimicrobial compound acrolein, which is generated from the metabolic intermediate β-hydroxy-propionaldehyde (Engels et al., 2016b).

Since the reduction of β-hydroxy-propionldehyde is dependent on the availability of reduced co-factors (Gänzle, 2015; Dishisha et al., 2019), the differential growth rates that we observed with glucose, maltose, lactose and raffinose likely relate to the differential reuterin accumulation. Rapid growth and metabolism of maltose and glucose provides reduced co-factors that rapidly convert β-hydroxy-propionldehyde to 1,3-propanediol, while slow metabolism of lactose via the Leloir pathway also reduces the supply of NADH and hence favors reuterin accumulation (Zhao and Gänzle, 2018). The galactose moiety of raffinose is also metabolized via the Leloir pathway but raffinose additionally supplies fructose as an alternative electron acceptor that competes with β-hydroxy-propionaldehyde as electron acceptor (Gänzle, 2015). The availability of glycerol in the large intestine is likely much lower when compared to the upper intestine, although the production of acrolein by intestinal microbiota demonstrates that the *pdu* pathway is active in the large intestine (Zhang J. et al., 2018). The large intestine is also characterized by a paucity of fermentable carbohydrates as digestible sugars are absorbed by the host while (conditionally) indigestible oligosaccharides including raffinose and lactose are rapidly fermented by intestinal microbiota in the terminal ileum (Zoetendal et al., 2012). Thus, the contribution of the pathway to overall metabolism and ecological fitness is dependent on the site of metabolism.

Reuterin inhibition assays confirmed that reuterin produced by *L. reuteri* is sufficient to inhibit the intestinal bacterium *E. coli*, showing that reuterin can be produced at concentrations that are ecologically relevant. These results agree with previous findings that reuterin formed by *L. reuteri* under glycerol supplementation is capable of inhibiting some enteric bacteria, e.g., *Listeria monocytogenes*, *Helicobacter pylori*, *Salmonella Typhimurium* (Cleusix et al., 2007; De Weirdt et al., 2012; Langa et al., 2018; Urrutia-Baca et al., 2018). Additionally, reuterin biosynthesis by *L. reuteri* in the gut environment was observed (Morita et al., 2008; Spinler et al., 2017), which supports the view that reuterin acted as an antibacterial agent for competition and fitness in the gut and suggests that reuterin formation is one of the traits encoded by the *pdu* cluster that are shaped through the evolution of *L. reuteri* (Walter et al., 2011; Zhang J. et al., 2018). However, our results reveal that *L. reuteri* strains capable of reuterin formation do not necessarily relate to a higher resistance towards reuterin. For example, although DSM 17938 is a far superior reuterin producer, it was much more sensitive towards reuterin than ATCC 6475. These findings suggest that the concentration of reuterin *in vivo* was not high enough to generate a selective pressure to evolve resistance. Therefore, questions remain on the ecological and evolutionary roles of reuterin formation, and its importance for maintaining the *pdu* cluster in *L. reuteri* strains despite its burden for the fitness of *L. reuteri in vivo* (Cheng et al., 2020).

Our findings on the substrate-dependent regulation of the *pdu* cluster functions are relevant for our understanding of the ecological roles of *L. reuteri* in their respective hosts. Lineage II strains produce the highest levels of reuterin on raffinose and lactose, which are dominant in the gut of human adults and infants, respectively. Such findings may suggest an adaptation of the *pdu* cluster to maximize reuterin formation for antagonistic competition in such environments. In contrast, phylogenetic, genomic, and functional (through competition experiments) analyses indicate an adaptation of Lineage VI strains to birds (Duar et al., 2017), where they colonize the crop. The crop is a nutrient-rich environment where neither carbon sources nor alternative electron acceptors are limiting factors, and sugars like glucose and maltose are likely to be present in abundance. However, the bacteria in the crop are constantly challenged by competition with feed-derived microorganisms. The high growth rates of lineage VI strains on glucose and maltose, concurrent with the lack of repression on reuterin formation with these substrates, might constitute an adaptation to colonize the crop environment. A “leaky” reduction of the noxious metabolic intermediate β-hydroxy-propionaldehyde may support competitiveness after conversion to acrolein, inhibiting competitors. Overall, it seems that different lineages fine-tune the regulation of *pdu* operon to either maximize or sacrifice metabolic efficiency in favor of accumulating an antimicrobial intermediate that inhibits competitors.

Although our present study revealed novel information on the phenotypic regulation of the *pdu* cluster allowing for interpretations of its role for *L. reuteri* ecology and evolution, important questions remain to be answered. Being focused on a phenotypic characterization of the *pdu* cluster in a phylogenetic context, our study did not address the molecular, biochemical, and metabolic basis of our findings. To gain such mechanistic insight, future studies should characterize gene expression within the *pdu* cluster during growth with the carbohydrates used above in different *L. reuteri* lineages in extension to what has been done by Spinler et al. (Spinler et al., 2014). In addition, the genetic and biomedical mechanisms by which carbohydrate metabolism differs between *L. reuteri* lineages should be elucidated. Moreover, although our findings provide important basic information for the generation of hypotheses on the ecological role of *pdu* cluster, these hypotheses should be evaluated through *in vivo* experiments in relevant host systems using experimental designs similar to those utilized in our previous studies (Duar et al., 2017; Lin et al., 2018; Cheng et al., 2020). Finally, *pdu* cluster functionality should be tested with additional carbohydrates that are therapeutically relevant, such as prebiotics (Rattanaprasert et al., 2019).

Apart from offering insight into the *pdu* cluster related functions that might influence the ecology of *L. reuteri*, our findings are also relevant for its biotechnological use to commercially produce reuterin, which could be improved by using substrates such as raffinose. In addition, therapeutic applications of *L. reuteri* could be improved by using raffinose and lactose in more targeted synbiotic combinations for human adults (raffinose) and infants (lactose) to potentiate the probiotic strain’s competitiveness and antimicrobial effects.

## Data Availability Statement

The raw data supporting the conclusions of this article will be made available by the authors, without undue reservation.

## Author Contributions

ZZ and KW performed the experiments and analyzed the data. ZZ wrote the first draft of manuscript with contributions from KW. CC and DR provided critical discussion of the data and contributed to experiments. HW co-supervised ZZ and gave advice for manuscript preparation. J-HO, SZ, and J-PP provided the mutants. MG and JW designed the project, supervised research, and wrote the manuscript. All authors contributed to the article and approved the submitted version.

## Conflict of Interest

The authors declare that the research was conducted in the absence of any commercial or financial relationships that could be construed as a potential conflict of interest.
