# Supplementary material for: A Phylogenetic View on the Role of Glycerol for Growth Enhancement and Reuterin Formation in Limosilactobacillus reuteri
Source: Front Microbiol. 2020 Dec 21;11:601422. doi: 10.3389/fmicb.2020.601422 (PMC7779471; doi:10.3389/fmicb.2020.601422)
Supplement: Supplementary file 1 [file Table_1.DOC]

**FIGURE S1** Calibration curve of the proposed acrolein used for reuterin quantification. Experiments were performed in triplicate.

Figure S1

**FIGURE S2** Representative images of reuterin formation and inhibition for *E. coli* without glycerol utilization. Overnight cultures of lineage II human strain ATCC 6475, lineage VI strain DSM 17938 and their mutant strains ATCC 6475∆*pduCDE* and DSM 17938∆*pduC* were spotted on basal MRS with 1.5% carbohydrate, *E. coli* strains in LB soft agar with 1.0% carbohydrate but without glycerol were overlaid on *L. reuteri* spots.

Figure S2

**FIGURE S3** The agar spot overlay assay for *E. coli* inhibition by *L. reuteri* in vitro. Overnight lineage II strain ATCC 6475, lineage VI strain DSM 17938 and their mutant strains were spotted on basal MRS with 1.0% carbohydrate, *E. coli* strains in LB soft agar containing 300 mM glycerol with 1.0% carbohydrate were overlaid on *L. reuteri* spots. **(A)** Representative images of Reuterin formation and inhibition for *E. coli* from four carbohydrates. **(B)** Halo sizes of inhibition measurements for *L. reuteri* strains used against *E. coli*. Data for the same strain grown on different sugars differ significantly (*p* < 0.05) if bars don’t share a common superscript.

Figure S3

**FIGURE S4** Representative images of reuterin formation and inhibition for *E. coli* without glycerol utilization. Overnight cultures of lineage II human strain ATCC 6475, lineage VI strain DSM 17938 and their mutant strains ATCC 6475∆*pduCDE* and DSM 17938∆*pduC* were spotted on basal MRS with 1.0% carbohydrate, *E. coli* strains in LB soft agar with 1.0% carbohydrate but without glycerol were overlaid on *L. reuteri* spots.

Figure S4
